# Primitive neuroectodermal tumour of the kidney with vena caval and atrial tumour thrombus: a case report

**DOI:** 10.1186/1752-1947-2-265

**Published:** 2008-08-11

**Authors:** Poh Ho Ong, Ramaswamy Manikandan, Joe Philip, Kirsten Hope, Michael Williamson

**Affiliations:** 1 Department of Urology, University Hospital Aintree, Liverpool, L9 7AL, UK; 2 Department of Pathology, Royal Liverpool University Hospital, Liverpool, L7 8XP, UK

## Abstract

**Introduction:**

Renal primitive neuroectodermal tumour is an extremely rare malignancy.

**Case presentation:**

A 21-year-old woman presented with microscopic haematuria, a palpable right loin mass, dyspnoea, dizziness and fatigue. Initial ultrasound scan of the kidneys revealed an 11 cm right renal mass with venous extension into the inferior vena cava. Computed tomography of the thorax and abdomen revealed an extension of the large renal mass into the right renal vein, inferior vena cava and up to the right atrium. A small paracaval lymph node was noted and three small metastatic nodules were identified within the lung parenchyma. The patient underwent a radical nephrectomy and inferior vena caval tumour (level IV) thrombectomy with cardiopulmonary bypass and deep hypothermic circulatory arrest. Immunohistochemical staining of the specimen showed a highly specific cluster of differentiation (CD) 99, thus confirming the diagnosis of a primitive neuroectodermal tumour.

**Conclusion:**

It is important that a renal primitive neuroectodermal tumour be considered, particularly in young patients with a renal mass and extensive thrombus.

## Introduction

Primitive neuroectodermal tumour (PNET) of the kidney is an extremely rare malignancy. Renal PNET is highly aggressive presenting at an advanced stage with metastasis and subsequent poor prognosis. It affects young adults with significant mortality owing to the late diagnosis, advanced stage and aggressive course of the disease [1,2]. We report a case of a primary renal PNET with extensive inferior vena caval and atrial tumour thrombus and with multiple lung metastases.

## Case presentation

A 21-year-old woman was referred with an occasionally painful right loin mass, persistent microscopic haematuria and lower urinary tract symptoms of 3-month duration. She reported increasing breathlessness and felt dizzy whilst carrying out routine activities. Clinical examination revealed only a weak radial pulse and a palpable right renal mass with no ascites or peripheral oedema.

**Figure 1:**
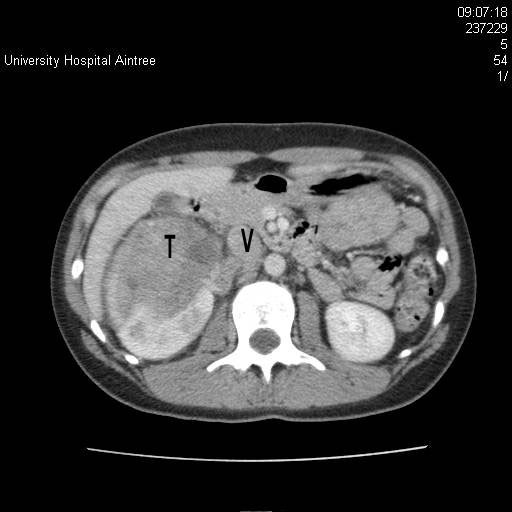
Computed tomography scan showing a large right renal tumour (T) extending into the renal vein and inferior vena cava (V).

**Figure 2:**
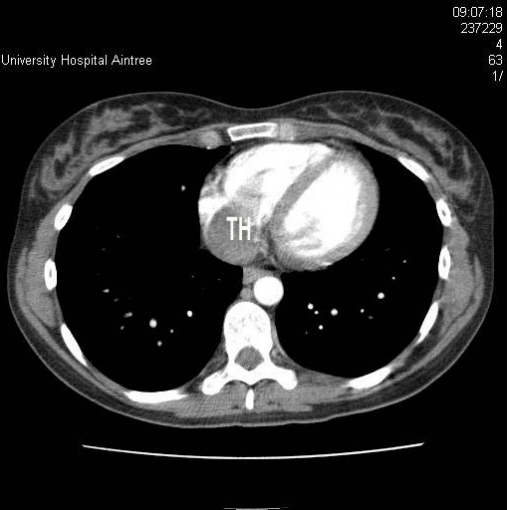
**Computed tomography scan of the chest showing the tumour thrombus (TH) in the right atrium**.

Ultrasound scan (USS) revealed a large 11 cm mass arising from the lower aspect of the right kidney, which extended along the right renal vein and into the inferior vena cava (IVC) and up to the diaphragm. Further radiological studies included magnetic resonance imaging (MRI) and computed tomography (CT) of the thorax and the abdomen. MRI of the kidney revealed a large 13 cm, right encapsulated, lower-aspect renal mass with tumour thrombus extending into the right renal vein, IVC and into the right atrium and occupying a significant proportion of the right atrial volume. The right atrium appeared largely distended with thrombus with a faint trickle of contrast just getting past its wall (Figures 1 and 2). There was a 12 mm paracaval lymph node and increased vascularity in the adjacent perinephric bed. Three nodules of less than 5 mm were identified within the lung parenchyma.

The patient underwent right radical nephrectomy and IVC and atrial tumour (level IV) thrombectomy with cardiopulmonary bypass in deep hypothermic circulatory arrest. The postoperative period was unremarkable apart from a pericardial effusion, which was aspirated.

**Figure 3:**
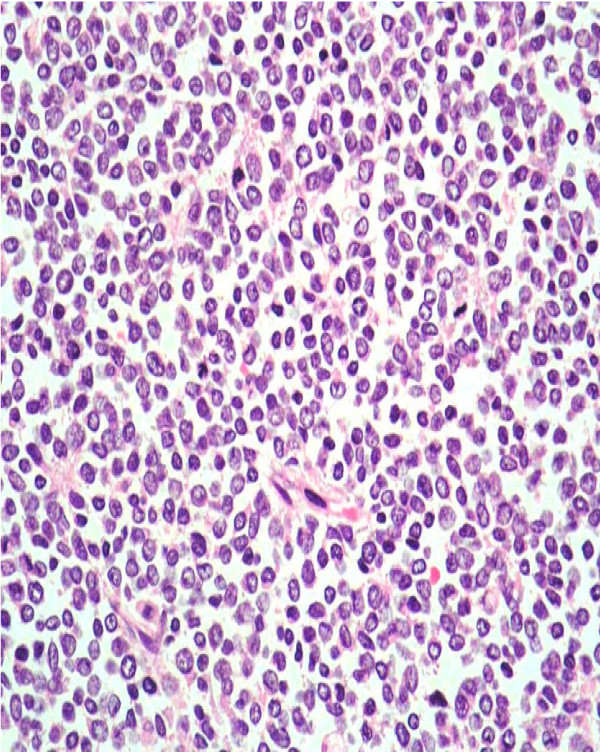
**Histology of the tumour**. Cohesive sheets of small, uniform, primitive, blastema-like malignant cells are separated by fibrous bands. Perivascular rosetting was seen but there was no architectural arrangement (haematoxylin and eosin, magnification ×20).

Gross examination revealed a friable, greyish white, lobulated mass (125 mm × 90 mm), which replaced most of the kidney with only a small amount of uninvolved parenchyma at the lower pole. Haematoxylin and eosin staining showed the tumour to comprise cohesive sheets of small, uniform, primitive, blastema-like malignant cells separated by fibrous bands. Perivascular rosetting was noted, but there was no architectural arrangement. The malignant cells had only a small amount of cytoplasm, and there was brisk mitotic activity (Figure 3). The tumour also infiltrated the IVC.

**Figure 4:**
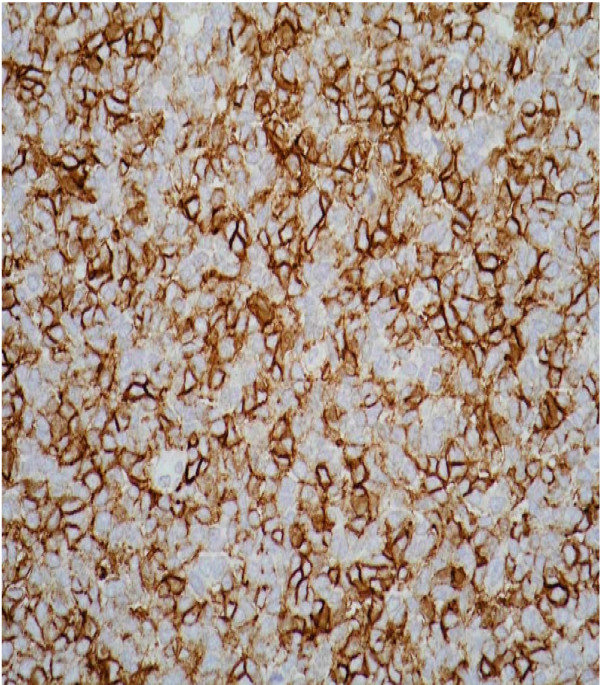
Immunohistochemical staining showing a diffuse expression of the cluster of differentiation 99 antigen.

The differential diagnosis was blastema-predominant Wilms' tumour and a peripheral PNET. Immunohistochemical staining exhibited diffuse expression of the cluster of differentiation (CD) 99 (Figure 4) and CD56 antigens, but not the Wilms' tumour suppressor gene (*WT1*), indicating PNET as the most likely diagnosis.

Prechemotherapy CT scans of the thorax, abdomen and pelvis demonstrated no evidence of residual or local recurrent disease. There was no lymphadenopathy or evidence of pulmonary abnormality. There was thrombus in the IVC extending as far as the right atrium. A whole body bone scan was negative. The patient then underwent eight cycles of adjuvant chemotherapy (vincristine, ifosfamide, doxorubicin and etoposide).

A CT scan after 4 months showed regression of the pulmonary nodules. The patient remained well at a 10-month follow-up.

## Discussion

Primitive neuroectodermal tumour of the kidney tends to affect young adults with no gender preponderance. It is a rare tumour with about 200 reported cases in the literature [1-3]. However, the exact number of cases could be difficult to determine as the tumours may not be clearly differentiated from extraskeletal Ewing's sarcoma. Patients typically present with haematuria, a palpable abdominal mass and flank and/or abdominal pain [4-8]. Our patient, with extensive atrial thrombus, complained of dyspnoea, dizziness and fatigue owing to the mechanical effect of the tumour in the right atrium causing circulatory compromise.

Diagnosing PNET can be challenging as it is sometimes difficult to differentiate it from other primary renal neoplasms, such as Wilms' tumour. Macroscopically they are bulky tumours. They tend to be greyish in colour, encapsulated and contain focal areas of haemorrhage and/or necrosis. The tumour is usually sharply demarcated from a normal kidney. Classically, a PNET histologically shows small round cells and may form several neuroblastic Homer Wright rosettes, or pseudorosettes.

In this case, the clinical diagnosis was of a renal cell carcinoma. The diagnosis was confirmed by positive immunohistochemical staining for CD99, but not *WT1*. Special stains and neural markers, such as CD99, neuron-specific enolase and monoclonal antibodies can help in making the correct diagnosis. CD56, also called a neural cell adhesion molecule, is a homophilic-binding glycoprotein expressed on the surface of neurons, glia, skeletal muscle and natural killer cells. Neuroendocrine and Wilms' tumours are CD56 positive, while PNET is usually CD56 negative. CD99 are cell-surface glycoproteins highly expressed on thymocytes, Ewing's sarcoma, PNET cells, pancreatic islet cells, Leydig and Sertoli cells and moderately on haematopoietic cells. Should these prove insufficient for establishing a diagnosis, electron microscopy, deoxyribose nucleic acid (DNA) image cytometry, fluorescent *in situ *hybridization and molecular pathology, such as reciprocal translocation of chromosomes 11 and 22 [t(11;22)(q24;q12)], can be used as confirmatory tests [3]. PNETs have a specific chromosomal translocation t(11; 22), which results in a chimeric EWS-FLI-1 that is a highly specific molecular marker for PNET.

Karnes et al. [6] reported, in 2000, the first case of a PNET with vena caval tumour thrombus (level II). Thomas et al. [7] first reported a PNET with a level IV thrombus in a 55-year-old woman, which was managed successfully with deep hypothermic circulatory arrest. This patient was 21 years old and one of the youngest patients with PNET with a level IV thrombus to undergo right radical nephrectomy and IVC tumour (level IV) thrombectomy with cardiopulmonary bypass and deep hypothermic circulatory arrest.

Chen et al. [4] reported the case of a 17-year-old woman with a right renal PNET, which extended into the vena cava, right atrium and hepatic veins. The patient had Budd Chiari syndrome and also underwent thrombectomy with cardiopulmonary bypass and deep hypothermic circulatory arrest. To date, there have only been two cases of Budd Chiari syndrome secondary to renal PNET [4].

Our patient had spontaneous regression of pulmonary metastases after nephrectomy similar to that described in Wada et al. [8]. To date, there is no absolute protocol or treatment for PNET owing to its rarity. Most reported cases underwent (radical) nephrectomy, adjuvant chemotherapy (vincristine, ifosfamide, doxorubicin, cyclophosphamide and etoposide), radiotherapy or bone marrow transplant. The prognosis of PNET remains poor despite these therapies [3-8]. Thyavihally et al. [3]-reported a 60% and 42% survival rate at 3 and 5 years, respectively. As illustrated in this case, it is important to consider the possibility of a renal PNET in young patients presenting with a renal mass and particularly those with extensive vena caval or atrial thrombus.

## Abbreviations

CD: Cluster of differentiation; CT: Computed tomography; DNA: Deoxyribose nucleic acid; IVC: Inferior vena cava; MRI: Magnetic resonance imaging; PNET: Primitive neuroectodermal tumour; USS: Ultrasound scan.

## Competing interests

The authors declare that they have no competing interests.

## Authors' contributions

PHO drafted the manuscript, prepared the illustrations and carried out the literature search. RM conceived the idea of the study, helped to draft the manuscript and helped to acquire the CT images. JP helped to draft the manuscript and with the literature search. KH helped to draft the manuscript, paying particular attention to the pathological aspects, and acquired the histological images for illustration. EPMW conceived of this study and supervised the drafting and overall structure of the manuscript. All the authors read and approved the final manuscript.

## Consent

Written informed consent was obtained from the patient for publication of this case report and accompanying images. A copy of the written consent is available for review by the Editor-in-Chief of this journal.
